# Comparative analysis of glutaredoxin domains from bacterial opportunistic pathogens

**DOI:** 10.1107/S1744309111012346

**Published:** 2011-08-16

**Authors:** Thomas Leeper, Suxin Zhang, Wesley C. Van Voorhis, Peter J. Myler, Gabriele Varani

**Affiliations:** a School of Medicine, University of Washington, Seattle, WA 98195, USA; b Seattle Structural Genomics Center for Infectious Disease (SSGCID), USA; c Seattle Biomedical Research Institute, 307 Westlake Avenue North, Suite 500, Seattle, WA 98109, USA; d Departments of Global Health and Medical Education and Biomedical Informatics, University of Washington, Seattle, WA 98195, USA

**Keywords:** glutaredoxins, metal detoxification, reactive oxygen species, ribonucleotide reductases, *Brucella melitensis*, *Bartonella henselae*, cat-scratch fever, Malta fever, thioredoxin fold

## Abstract

Glutaredoxin proteins (GLXRs) are essential components of the glutathione system that reductively detoxify substances such as arsenic and peroxides and are important in the synthesis of DNA *via* ribonucleotide reductases. NMR solution structures of glutaredoxin domains from two Gram-negative opportunistic pathogens, *Brucella melitensis* and *Bartonella henselae*, are presented. These domains lack the N-terminal helix that is frequently present in eukaryotic GLXRs. The conserved active-site cysteines adopt canonical proline/tyrosine-stabilized geometries. A difference in the angle of α-helix 2 relative to the β-­sheet surface and the presence of an extended loop in the human sequence suggests potential regulatory regions and/or protein–protein interaction motifs. This observation is consistent with mutations in this region that suppress defects in GLXR–ribonucleotide reductase interactions. These differences between the human and bacterial forms are adjacent to the dithiol active site and may permit species-selective drug design.

## Abstract 2

NMR structures of the glutaredoxin (GLXR) domains from *Br. melitensis* and *Ba. henselae* have been determined as part of the SSGCID initiative. Comparison of the domains with known structures reveals overall structural similarity between these proteins and previously determined *E. coli* GLXR structures, with minor changes associated with the position of helix 1 and with regions that diverge from similar structures found in the closest related human homolog.

## 1. Introduction

**Figure 1:**
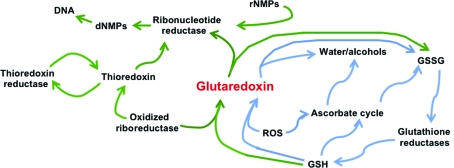
Diagram of the role that glutaredoxin and related enzymes play in DNA synthesis and ROS, metal and metalloid detoxification. Abbreviated pathways utilized by glutaredoxin and thioredoxin in DNA synthesis are highlighted in green; abbreviated ROS defense pathways are highlighted in blue.

Glutaredoxins (GLXRs) are redox enzymes that are important for the reduction of ribonucleotide reductase enzymes that synthesize deoxynucleotides from ribonucleotides (Uhlin & Eklund, 1994 ▶). Thus, they are required for efficient and sustainable synthesis of DNA. Additionally, GLXRs are important for detoxifying oxidizing agents such as reactive oxygen species (ROS), transition metals and metalloids, *e.g.* arsenic compounds (Fig. 1 ▶). Like other ROS defenses, *i.e.* glutathione peroxidases, this enzyme is connected to the glutathione pool: GLXRs catalyse the reaction of glutathione with peroxides and metals as shown in (1). Homeostatic levels of reduced glutathione are restored by the action of glutathione reductase (GSR) in (2) *via* reducing equivalents from the pentose phosphate shunt. Thus, the GLXR, glutathione peroxidase and glutaredoxin reductase enzymes are attractive targets for drug-mediated ROS amplification.
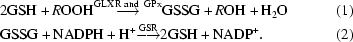
GLXRs have well conserved sequences within bacteria, but their sequences diverge between bacteria and humans. This distinctive difference in sequence should permit selective inhibition of bacterial GLXRs without perturbation of the host enzyme. This might kill bacteria by inhibition of DNA synthesis and/or through increases in ROS toxicity.

Structures have been published for several forms of human (Sun *et al.*, 1998 ▶; Yang *et al.*, 1998 ▶), plant (Rouhier *et al.*, 2007 ▶; Li *et al.*, 2010 ▶), budding yeast (Gibson *et al.*, 2008 ▶; Discola *et al.*, 2009 ▶) and *Escherichia coli* GLXRs (Iwema *et al.*, 2009 ▶; Fladvad *et al.*, 2005 ▶; Xia *et al.*, 1992 ▶, 2001 ▶; Bushweller *et al.*, 1994 ▶; Sodano *et al.*, 1991 ▶). However, it was unclear whether other bacterial GLXRs would adopt similar conformations. The aim of this study was to expand the existing knowledge base of GLXR structures and to find structural trends that might be exploited to design selective inhibitors of bacterial GLXR that leave host enzymes unperturbed. In particular, the GLXRs from the pathogens *Brucella melitensis* and *Bartonella henselae* were investigated as these organisms have significant relevance to medical and military biodefense. Here, we present the structures of GLXRs from *Br. melitensis* and *Ba. henselae* and compare these structures with the available structures from *E. coli* and human.

## 2. Methods

### 2.1. Protein expression and purification

GLXRs from *Br. melitensis* and *Ba. henselae* (NCBI YP_415222 and YP_033241.1; UniProt Q2YLN2 and Q6G5J5; Pfam ID PF00462; EC 1.20.4.1) were cloned into a pAVA vector (Choi *et al.*, 2011 ▶) and expressed from RIL cells grown in 2 l of M9 medium supplemented with 4 g l^−1^ ^13^C glucose and 1 g l^−1^ ^15^N ammonium chloride. Protein expression was induced at an OD_600_ of 0.6 with 0.5 m*M* IPTG and temperature reduction to 293 K for 12 h. Cell pellets were suspended in 50 ml buffer *A* (20 m*M* HEPES pH 7, 0.3 *M* NaCl, 5% glycerol, 2 m*M* DTT) supplemented with 2 µg lysozyme, freeze-fractured twice at 193 K and then lysed using a French press. The crude lysate was cleared by centrifugation at 15 000*g* and the soluble protein supernatant was filtered through a 0.22 µm GD/X membrane syringe filter (Whatman). Nickel IMAC on 5 ml HisTrap FF columns (GE Healthcare) was used to capture the proteins from this supernatant. Non­specific binding proteins were washed off the column with 10% buffer *B* (20 m*M* HEPES pH 7, 0.3 *M* NaCl, 5% glycerol, 2 m*M* DTT, 300 m*M* imidazole). The proteins were eluted with a 50 ml gradient from 10% to 100% buffer *B* and fractions containing purified protein were pooled, cleaved with 3C protease and rerun over the HisTrap column. The N-terminal tag introduced during cloning consisted of an MAHHHHHHMGTLEAQTQGPGS sequence appended to the native methionine; only the GPGS portion remained after protease cleavage. The HisTrap flowthroughs were collected, dialyzed against NMR buffer (20 m*M* phosphate, 120 m*M* NaCl pH 6) and purified by size-exclusion chromatography on a Superdex 75 column (GE Healthcare) equilibrated with NMR buffer. Fractions were pooled and concentrated *via* stirred cell (Amicon) to 0.5 m*M* for *Ba. henselae* GLXR and to 1.5 m*M* for *Br. melitensis* GLXR and placed in NMR microcells (Shigemi).

### 2.2. NMR data collection

For both proteins, the standard suite of NMR experiments were acquired (Sattler *et al.*, 1999 ▶): ^15^N HSQC, ^13^C HSQC, 3D HNCO, 3D HNCA, 3D HN(CO)CA, 3D CBCA(CO)NH, 3D HNCACB, 3D HCCH-TOCSY, 3D ^15^N-TOCSY-HSQC (70 ms mixing), 3D ^15^N and ^13^C NOESY-HSQC (80 and 120 ms mixing times) and 2D ^1^H/^1^H D_2_O-NOESY (100 ms mixing time). Two instruments were used for data collection: Bruker Avance 500 and 600 spectrometers equipped with cryoprobes. All data sets were collected in conventional, *i.e.* nonreduced dimensionality, formats with States–TPPI quadrature (States *et al.*, 1982 ▶) in the indirect ^13^C and ^1^H dimensions and Rance–Kay sensitivity enhancement (Kay *et al.*, 1992 ▶; Cavanagh *et al.*, 1991 ▶) for ^15^N dimensions. Proton carriers were set on water and the ^15^N carrier at 117 p.p.m. For α-carbon relevant spectra the ^13^C carrier was set to 52 p.p.m., while for CACB spectra it was set to 45 p.p.m. and for carbonyl spectra it was set to 176 p.p.m.. Spectra were referenced directly to DSS in proton dimensions and indirectly in ^13^C and ^15^N dimensions. NMR data sets were converted and processed with *NMRpipe* (Delaglio *et al.*, 1995 ▶).

### 2.3. Assignments and structure calculations

Backbone assignments for both proteins were determined from pairs of triple-resonance spectra in the usual manner (Sattler *et al.*, 1999 ▶; Lunde *et al.*, 2010 ▶; Leeper *et al.*, 2010 ▶). Backbone resonance correlations were compared and tabulated using *CCPNMR* (Vranken *et al.*, 2005 ▶) using the manual assignment mode. Side chains were assigned from HCCH-TOCSY, ^15^N-TOCSY-HSQC and, in the case of aromatic residues, a 2D ^1^H/^1^H D_2_O-NOESY. Distance constraints for structure calculations were obtained from 2D ^1^H/^1^H D_2_O-NOESY and 3D ^15^N and ^13^C NOESY-HSQC spectra as unassigned peak lists. Peak intensities were exported directly from these spectra for use in *CYANA* structure calculations (Güntert, 2004 ▶), as were chemical shifts for *TALOS*-generated dihedral angle restraints (Shen *et al.*, 2009 ▶). Hydrogen-bond constraints were determined for slowly D_2_O-exchangeable backbone amides with acceptor-atom identities gleaned from preliminary structure calculations. Initially, the disulfide bond in the active site was left as a pair of thiols, but was ultimately restrained to be a disulfide based upon initial structure geometry and proximity. We decided to use the structure calculations to guide this decision since these residues are helical and the C^β^ shifts reside in the ambiguous border region between 30 and 33 p.p.m.: normal C^β^ shifts for reduced helical thiols range from 23.8 to 28.8 p.p.m., but oxidized helical disulfide C^β^ atoms range from 32.8 to 47.4 p.p.m. (Sharma & Rajarathnam, 2000 ▶). Note that no particular effort was made to maintain this pair of cysteines in the reduced state, so they are likely to have been oxidized spontaneously. We have not yet explored thorough p*K*_a_ calculations to determine whether these cysteines exist as a mixed thiol/thiolate state (Sun *et al.*, 1998 ▶; Yang *et al.*, 1998 ▶), but we may do so in future studies.

Seven rounds of automated NOE assignment and structure calculation using *CYANA*’s *CANDID* tool (Herrmann *et al.*, 2002 ▶) were used to calculate the structures, followed by one round of manual calculation of 100 structures. The final ensembles were selected as the 20 structures with the lowest *CYANA* target functions. These structures showed convergence *via* low r.m.s.d.s (Table 1 ▶) and excellent covalent geometry and clash scores (Table 2 ▶) as determined by *MolProbity* (Chen *et al.*, 2010 ▶). Structure ensembles were analysed and rendered with *PyMOL* (DeLano & Lam, 2005 ▶).

## 3. Results and discussion

### 3.1. Sequence conservation between domains

**Figure 2:**
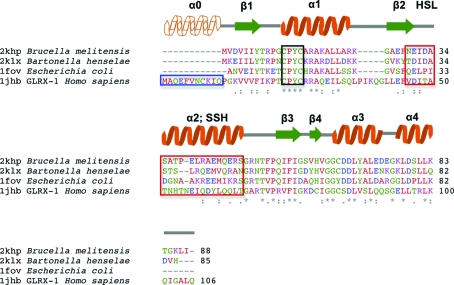
Multiple sequence alignment of the *Br. melitensis*, *Ba. henselae* and *E. coli* glutaredoxin 3 domains and the *Homo sapiens* glutaredoxin 1 domain. The black-boxed region indicates the conserved active-site residues shared with all dithiol GLXRs, while the red-boxed region highlights the human-specific loop 2 region (HSL2) and adjacent sequence-specific helix (SSH) region. The blue-boxed region is the additional N-terminal helix found in the human protein.

A *BLAST* search of *Br. melitensis* and *Ba. henselae* GLXR-domain sequences against the nonredundant protein database (Altschul *et al.*, 1990 ▶) revealed that the *E. coli* GLXR3 domain was the closest known homolog (59% identity, *E* value = 3 × 10^−20^ *versus* 2khp). Upon inspection of closest homologs from available human sequences, the GLXR1 sequence was revealed to be most similar to the bacterial GLXR3 (38% identity, *E* value = 1 × 10^−10^ *versus* 2khp). We assume that this represents a discrepancy in the annotation rather than a functional difference, as human GLXR3 is significantly less related (25% identity, *E* value = 2 × 10^−3^ *versus* 2khp). A *ClustalW* alignment of the sequences using the BLOSUM matrix (Henikoff *et al.*, 1999 ▶; Larkin *et al.*, 2007 ▶) is shown in Fig. 2 ▶. From this comparison it is clear that for these sequences the region surrounding the redox active site is highly conserved (yellow box). There are very few overall differences between the new bacterial GLXR3s and the *E. coli* GLXR. However, when compared with the human GLXR1 sequence deviations are present in an N-terminal extension (α0), an inserted region in loop 1 between helix 1 and β-strand 2, and variations in the sequence of the loop between strand 2 and helix 2 and the N-terminal end of helix 2 are observed. As shown below, this last region is juxtaposed with the active site. As a result, we will refer to these latter two points of variation as the human-specific loop (HSL) and the sequence-specific helix (SSH), respectively.

### 3.2. Structures of glutaredoxin from *Br. melitensis* and *Ba. henselae*

**Figure 3:**
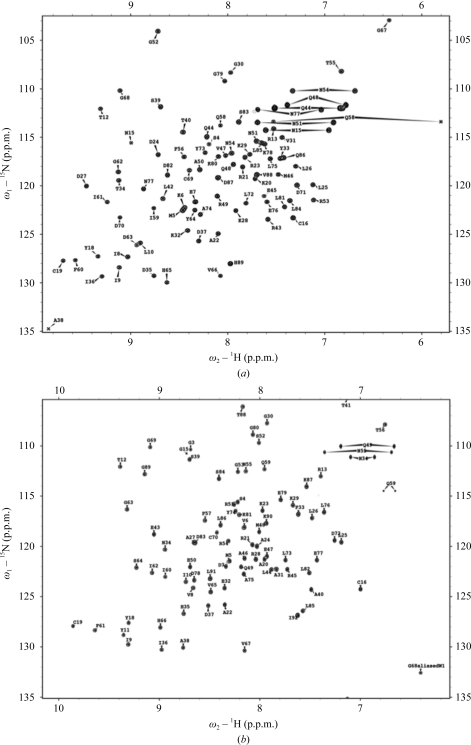
^15^N HSQC spectra of the *Ba. henselae* (*a*) and *Br. melitensis* (*b*) GLXR domains with complete backbone and side-chain amide assignments labeled.

NMR spectroscopy of the *Br. melitensis* and *Ba. henselae* GLXR domains revealed reasonably well resolved spectra that were amenable for structural study by NMR (Fig. 3 ▶). The *Br. melitensis* GLXR had a significantly larger number of unambiguously assignable NOEs than the *Ba. henselae* GLXR (Table 1 ▶). This is partially attributed to significantly stronger sample concentrations for the former (1.5 *versus* 0.5 m*M*), which are a result of a slight aggregation of the latter at higher concentrations as well as lower expression yields. Thus, the significantly larger numbers of medium and long-range constraints, which are also typically low signal-to-noise NOEs, for the *Br. melitensis* protein are a consequence of its higher concentration and improved spectral quality. Furthermore, *Ba. henselae* GLXR has ∼11 overlapped residues in the ^15^N HSQC, whereas *Br. melitensis* GLXR only has between two and six overlapped amides depending upon the field at which the spectra are collected (Fig. 3 ▶), thus further reducing the number of unambiguously assignable resonances.

**Figure 4:**
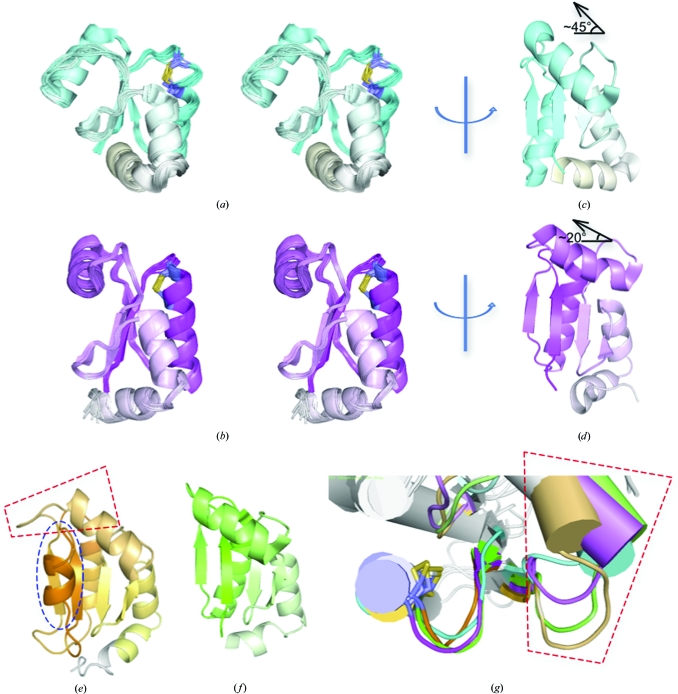
(*a*, *b*) Stereopairs of ensemble superposition for the NMR solution structures of the *Br. melitensis* (*a*) and *Ba. henselae* (*b*) glutaredoxins. The conserved pair of active-site cysteine side chains are drawn in gray and yellow. (*c*) The lowest energy conformer from (*a*) rotated to show the angle of SSH relative to the β-sheet surface. (*d*) The lowest energy conformer from (*b*) with a more parallel SSH angle. (*e*) The human glutaredoxin 1 structure with the extra N-terminal helix (blue) and the HSL/SSH region (red trapezoid) indicated. (*f*) The *E. coli* glutaredoxin 3 structure. (*g*) Close-up view of the convergent superposition of the CPYC active-site region (left) juxtaposed with the divergent and non-overlapping structures for the HSL/SSH regions. Only the region near the active site and the HSL/SSH regions are colored according to the above figures, while the remainder of the proteins are drawn in white.

Structure calculations for both the *Br. melitensis* and *Ba. henselae* GLXR domains converged well (Table 1 ▶, Figs. 4 ▶ *a* and 4 ▶ *b*). Topo­logically, these domains adhere to the expected thioredoxin fold: βαβαββαα with a 2134 mixed parallel and antiparallel β-sheet with helices on both sides of the sheet. The active-site CPYC residues are in the expected location at the N-terminal end of helix α1. The N- and C-terminal tails of these full-length domains are somewhat short relative to many other proteins studied by NMR, resulting in a well defined backbone conformation over the entire domain (0.52 and 0.35 Å r.m.s.d. over all backbone atoms including the N- and C-­termini). The Ramachandran statistics and *MolProbity* scores are good (Table 2 ▶) and suggest a well refined structure in spite of the heavy reliance upon the *CANDID* automated NOE assignment.

### 3.3. Comparison with other glutaredoxins: *E. coli* and human

The lowest energy structures for the *Br. melitensis* and *Ba. henselae* GLXR domains were compared with structures obtained for human GLXR1 (Fig. 4 ▶ *e*) and *E. coli* GLXR3 (Fig. 4 ▶ *f*). The most obvious difference is the presence of an extra N-terminal helix associated with the human domain (blue oval, Fig. 4 ▶ *e*). On further inspection, slight deviations in the angle of the SSH region also become apparent. In the *Ba. henselae* GLXR the SSH helical angle relative to the vector perpendicular to the β-sheet is about 45° (Fig. 4 ▶ *c*). This angle is similar to that of the human GLXR, which is sterically packed up against the C-terminal helical extension. In contrast, the *Br. melitensis* species-specific helix is more reminiscent of the *E. coli* structure, with an angle of about 20°. Thus, the SSH seems to vary among species at the levels of both primary sequence and three-dimensional structure.

## 4. Discussion and conclusion

We have determined the NMR structures of the GLXR domains from the pathogenic organisms *Br. melitensis* and *Ba. henselae*. These structures are typical examples of the thioredoxin fold present in many dithiol reductase enzymes. Furthermore, subtle differences in the ribonucleotide reductase binding platform on the SSH and the extension of the HSL suggest possible routes for rational species-selective drug design. For example, mutation of the SSH in *E. coli* GLXR3 allows it to thrive even in the inviable background con­taining gene knockouts for thioredoxin 1, thioredoxin 2 and GLXR1 (Ortenberg *et al.*, 2004 ▶). This mutation of Met43 to valine, isoleucine or leucine in the SSH seems to exert the restoration of its viability *via* enhanced interactions with ribonucleotide reductase, consistent with studies on GLXR bound to model peptides that point to a direct interaction with the SSH (Berardi & Bushweller, 1999 ▶). *E. coli* GLXR residue Met43 is on the opposite side of the helix from the surface expected to directly interact with ribonucleotide reductase, which suggests that replacement by more hydrophobic residues may adjust the position of this helix relative to the adjacent β-­sheet. This result emphasizes that the manner in which the SSH lays across this GLXR β-sheet surface may be pertinent to interactions with ribonucleotide reductase, a detail that is also relevant to GLXR isoform and species substrate-specificity (Figs. 4 ▶ *c* and 4 ▶ *d*). Additionally, the expression levels of ribonucleotide reductase, thio­redoxin and GLXR are tightly regulated so as to maintain relative stoichiometries (Miranda-Vizuete *et al.*, 1996 ▶). Thus, structural biology, biochemistry and epigenetics all point to the position of the sequence-specific helix (SSH) being important for recruitment of ribonucleotide reductase. Whether this is through direct interactions between ribonucleotide reductase and the SSH or whether the SSH acts as a displaceable cover for interactions mediated by the nearby β-sheet will require additional experiments to determine fully.

Either GLXR or thioredoxin is required for cellular viability (Russel & Holmgren, 1988 ▶). Unlike thioredoxin, GLXR requires no accessory enzymatic component to regenerate itself directly. Instead, it relies directly upon the state of the glutathione pool (typically at ∼99% GSH *versus* ∼1% GSSG; Higashi *et al.*, 1985 ▶) and hence the availability of reducing equivalents in the form of NADPH. Therefore, as a simpler molecular system, it may be more difficult to develop resistance pathways beyond the inherent alternative pathway provided by thioredoxin. Indeed, small-molecule inhibitors of glutathione synthesis such as buthione sulfoximine (BSO) can reverse resistance to cellular toxins and stress (Griffith & Meister, 1979 ▶). For example, both tumor cells and Gram-negative facultative anaerobic bacteria are highly dependent on the glutathione pool for viability (Smirnova *et al.*, 2005 ▶). It has been demonstrated that tumor cells that are resistant to radiation and chemotherapeutics can be sensitized *via* co-treatment with GSH synthesis inhibitors such as BSO. In a similar fashion, depletion of the glutathione pool using BSO-like compounds should amplify the effects of drugs targeting the GLXR in specific bacteria, although BSO itself has been shown to be only weakly effective against some strains of *E. coli* (Romero & Canada, 1991 ▶). Thioredoxin, on the other hand, senses the NADPH pool directly. Synthetic inhibition of thioredoxin and NADPH production might also be possible, since mutations in glucose-6-phosphate dehydro­genase, *i.e.* favism, are tolerated in the absence of ROS stress (Scriver, 2001 ▶). Thus, toxic side effects might be minimized for the host organism *via* direct inhibition of both thioredoxin and glucose-6-­phosphate dehydrogenase should that route be taken.

**Figure 5:**
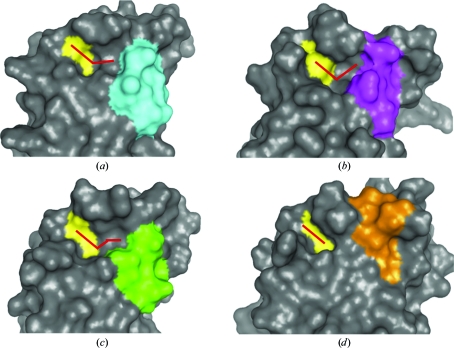
Surface renderings of the *Br. melitensis* (*a*), *Ba. henselae* (*b*), *E. coli* (*c*) and human (*d*) GLXR domains. The conserved disulfide active site is shown in yellow and the HSL loop regions are shown in cyan, magenta, green and gold as in Fig. 4 ▶. The V-shaped pocket amenable to drug design in the bacterial GLXRs is indicated by the red chevron. The protein poses are rotated 90° relative to Fig. 4 ▶(*g*).

GLXR and thioredoxin are nonspecifically inhibited by cisplatin (Arnér *et al.*, 2001 ▶) and cadmium (Chrestensen *et al.*, 2000 ▶). Additionally, glutathione analogs are also potent but nonsequence-specific inhibitors of GLXR (Höög *et al.*, 1982 ▶). Because these compounds are just as likely to disrupt host GLXRs as bacterial enzymes, they are not viable as drug candidates. Thus, the real challenge in finding dithiol active-site inhibitors lies in identifying compounds that disrupt or covalently react with the dithiol center but only after interrogating species-specific features. The relatively close proximity of the HSL region (Fig. 4 ▶ *g*, trapezoid) to the conserved active site affords a promising option. Surface renderings of the proteins support this assertion and highlight a V-shaped indentation bordered on one side by the conserved dithiol and on the other by the HSL (Figs. 5 ▶ *a*, 5 ▶ *b* and 5 ▶ *c*). This groove is much smaller within the surface of the human GLXR (Fig. 5 ▶ *d*). Thus, it may be possible to rationally engineer bidentate drugs that anchor themselves into the region *via* the HSL by one epitope while attacking the adjacent dithiol with their other halves. In the case of GLXR, such drugs would be particularly useful if combined with the aforementioned BSO compound for perturbing the basal GSH and/or NADPH levels to enhance ROS-mediated cell death.

## Supplementary Material

PDB reference: BrabA.00079.a, 2khp

PDB reference: BaheA.00334.a, 2klx

## Figures and Tables

**Table 1:** NMR restraints

	*Ba. henselae* (2klx)	*Br. melitensis* (2khp)
Distance restraints	1107	1747
Short range, |*i* − *j*| ≤ 1	621	886
Medium range, 1 < |*i* − *j*| < 5	207	374
Long range, |*i* − *j*| ≥ 5	279	487
Dihedral	202	194
Hydrogen bonds	40	22
*CYANA* target function (Å^2^)	1.45	1.11
Dihedral r.m.s.d. (°)	0.76	0.94
Distance r.m.s.d. (Å)	0.012	0.006
Maximum NOE violation (Å)	0.24	0.34

**Table 2:** Ensemble statistics Ensemble of 20 structures from 100 calculated structures.

	*Ba. henselae* (2klx)	*Br. melitensis* (2khp)
Backbone r.m.s.d. (mean) (Å)	0.52†	0.35‡
Heavy-atom r.m.s.d. (mean) (Å)	1.14†	0.82‡
Most favored (%)	83.8	90.8
Additionally allowed (%)	16.1	9.2
Generously allowed (%)	0.1	0.0
Disallowed (%)	0.0	0.0
*MolProbity* score (percentile)	3.57 (97th)	2.2 (99th)

† R.m.s.d. calculated over residues 3–84, excluding 1–2 and 85–89.

‡ R.m.s.d. calculated over residues 6–92, excluding 1–5.
